# Hif1α-dependent hypoxia signaling contributes to the survival of deep-layer neurons and cortex formation in a mouse model

**DOI:** 10.1186/s13041-022-00911-0

**Published:** 2022-03-31

**Authors:** Daisuke Sakai, Takeru Sugawara, Tomonori Kurokawa, Yuki Murakami, Mitsuhiro Tomosugi, Hiroko Masuta, Hiromi Sakata-Haga, Toshihisa Hatta, Hiroki Shoji

**Affiliations:** 1 Department of Biology, Kanazawa Medical University, 1-1 Daigaku, Uchinada, Ishikawa 920-0293 Japan; 2 Department of Medical Life Systems, Doshisha University, Kyotanabe, Kyoto 610-0394 Japan; 3 Department of Hygiene and Public Health, Kansai Medical University, Osaka, Hirakata 573-1010 Japan; 4 Department of Anatomy, Kanazawa Medical University, Uchinada, Ishikawa 920-0293 Japan

**Keywords:** Hif1α, Hypoxia, Cortex, Telencephalon, Mouse

## Abstract

Hypoxia-inducible factor 1 α (Hif1α) plays a crucial role in brain development. To study the function of Hif1α in early brain development, we generated neuroepithelial cell-specific *Hif1α*-knockout mice. *Hif1α*-knockout mice died soon after birth; these mice exhibited an abnormal head shape, indicating the presence of brain defects. Morphological analysis revealed that *Hif1α* ablation reduced the overall size of the brain, especially affecting the telencephalon. Neuronal apoptosis predominantly occurred in deep-layer neurons, consequently the alignment of cortical layers was severely disorganized in *Hif1α* knockout mice. Furthermore, we demonstrated that Vegf signaling contributes to the survival of deep-layer neurons as a downstream effector of Hif1α-dependent hypoxia signaling. Taken together, our findings demonstrate that Hif1α plays a critical role in the early stages of telencephalon development.

**Supplementary Information:**

The online version contains supplementary material available at 10.1186/s13041-022-00911-0.

## Introduction

In placental mammals, the embryo encounters drastic changes in the environmental oxygen concentration during development [1–4]. Even after the supply of oxygen from maternal blood, embryonic tissues, such as the neural tube, heart primordia, and intersomitic mesenchyme, continue to experience hypoxia [5, 6]. Proper development of mammalian embryos requires their adaptation to drastic changes in oxygen concentration. Therefore, mammalian embryos employ unique molecular pathways to enable gastrulation and organogenesis within a transient hypoxic environment. Indeed, it has been reported that culturing rodent embryos *exo utero* under hyperoxic conditions leads to abnormal embryo morphology [7].

Hypoxia-inducible factor 1 α (Hif1α), a key transcription factor that forms a complex with Hif1β, is involved in the cellular response to anaerobic conditions [8–11]. The stability of the Hif1α–Hif1β complex is regulated by the enzyme prolyl hydroxylase 1–3 (PHD1-3) in an oxygen-dependent manner. Under anaerobic conditions, the Hif1α–Hif1β complex is not hydroxylated, and therefore, is not targeted for degradation. The stabilized Hif1α–Hif1β complex binds to the hypoxia response element (HRE) to activate the expression of genes involved in energy metabolism, erythropoiesis, and angiogenesis, thereby protecting cells and tissues from hypoxic stress [12, 13]. In mammalian embryos, a Hif1α-dependent hypoxic response is required not only for the prevention of damage caused by hypoxic stress but also for proper progression of embryonic development. Hif1α-deficient mouse embryos exhibit defects in cardiovascular formation, somitogenesis, and neural tube closure, resulting in developmental arrest and lethality by embryonic day (E) 11 [14–17]. Furthermore, studies have shown that Hif1α is required for the formation of the heart, cartilage, and limbs, using conditional *Hif1α*–knockout mice [18–20]. Regarding the central nervous system (CNS), Christian et al*.* demonstrated that the ablation of *Hif1α* using the *Emx1-Cre* driver causes apoptosis of neurons and cortical hypoplasia in the cerebral cortex [21]. However, the function of *Hif1α* in cortical development has not been examined in detail. *Hif1α*-knockout mice have been generated using *Nestin-Cre* driver to observe a more broad-spectrum effect on neural development. Studies using *Nestin-Cre* driver have revealed that the ablation of *Hif1α* disrupts angiogenesis and consequently, expands a hypoxic region in the telencephalon at E16 [22]. Neural cell-specific ablation did not affect brain development until E15, but caused massive neuronal apoptosis in the telencephalon at E19, resulting in hydrocephalus at postnatal day (P) 70 [23]. Although *Nestin-Cre* mice express *Cre* mRNA throughout the CNS at E11.5, it is uncertain whether Cre is expressed before E11.5 [24]. In addition, Cre recombination activity is not detected in the ventricular zone (VZ) and subventricular zone (SVZ) at E12.5 and E14.5 in the commercially available *Nestin-Cre* line. Therefore, the *Nestin-Cre* line may be inefficient as a *Cre*-driver for recombination in embryonic neural progenitor cells [25]. Therefore, novel functions of Hif1α in early brain development could be discovered using other *Cre*-driver mice that express Cre within embryonic neural progenitor cells before E11.5. Herein, we investigated the function of Hif1α by neuroepithelial cell-specific ablation of *Hif1α* using *Sox1-Cre* [26]. In the *Sox1-Cre* driver, Cre activity was observed in neuroepithelial cells at least from E8.5; thus, the crossing of *Hif1α-*floxed mice with *Sox1-Cre* mice allow us to analyze the function of Hif1α in early neural development. In the present study, we demonstrated that *Hif1α* is required for neuronal survival, thereby facilitating the formation of cortical layers during telencephalon development.

## Results

### Generation of neuroepithelial cell-specific *Hif1α-*knockout mice

To address the biological significance of hypoxia in early brain development, we focused on deleting *Hif1α*, a master regulator of cellular response to hypoxia [8–11]. We crossed *Hif1α *^*flox/flox*^ mice [16] with *Sox1-Cre*^+/−^ mice [26] to selectively ablate *Hif1α* in early neural progenitor cells, thereby affecting all CNS cells. The resulting *Hif1α* gene carrying an exon 2 deletion encoded a malfunctioning Hif1α protein that lost the ability to induce the expression of genes containing HRE [16]. We confirmed the tissue specificity of Cre expression driven by *Sox1* promoter by generating *Sox1-Cre*^+/−^;* ROSA26/CAG-floxed STOP-tdTomato*^+/−^ reporter mice. Cre expression was readily detected by the red fluorescent signal in developing cortical neurons, but not in vasculature at E16.5, indicating neuroepithelial cell-specific expression of Cre driven by *Sox1* promoter (Additional file 1: Figure S1). Thus, we investigated the function of Hif1α-dependent hypoxic response in neural progenitor cells and their progeny during brain development. Heterozygotes (*Hif1α *^*flox/wt*^; *Sox1-Cre*^+/−^) were viable and fertile, indicating that ablation of a single *Hif1α* allele or the expression of Cre did not affect CNS development. In contrast, homozygotes (*Hif1α *^*flox/flox*^; *Sox1-Cre*^+/−^) conditional mutants died within several hours after birth*.* Mice homozygous for the floxed *Hif1α* allele with a *Sox1-Cre* allele (*Hif1α *^*flox/flox*^; *Sox1-Cre*^+/−^) were used to generate conditional knockout mice, which are hereafter referred to as “KO” mice. Mice homozygous for the floxed *Hif1α* allele without a *Sox1-Cre* allele (*Hif1α *^*flox/flox*^; *Sox1-Cre*^−/−^) were used as controls and are hereafter referred to as “WT” mice.

**Fig. 1:**
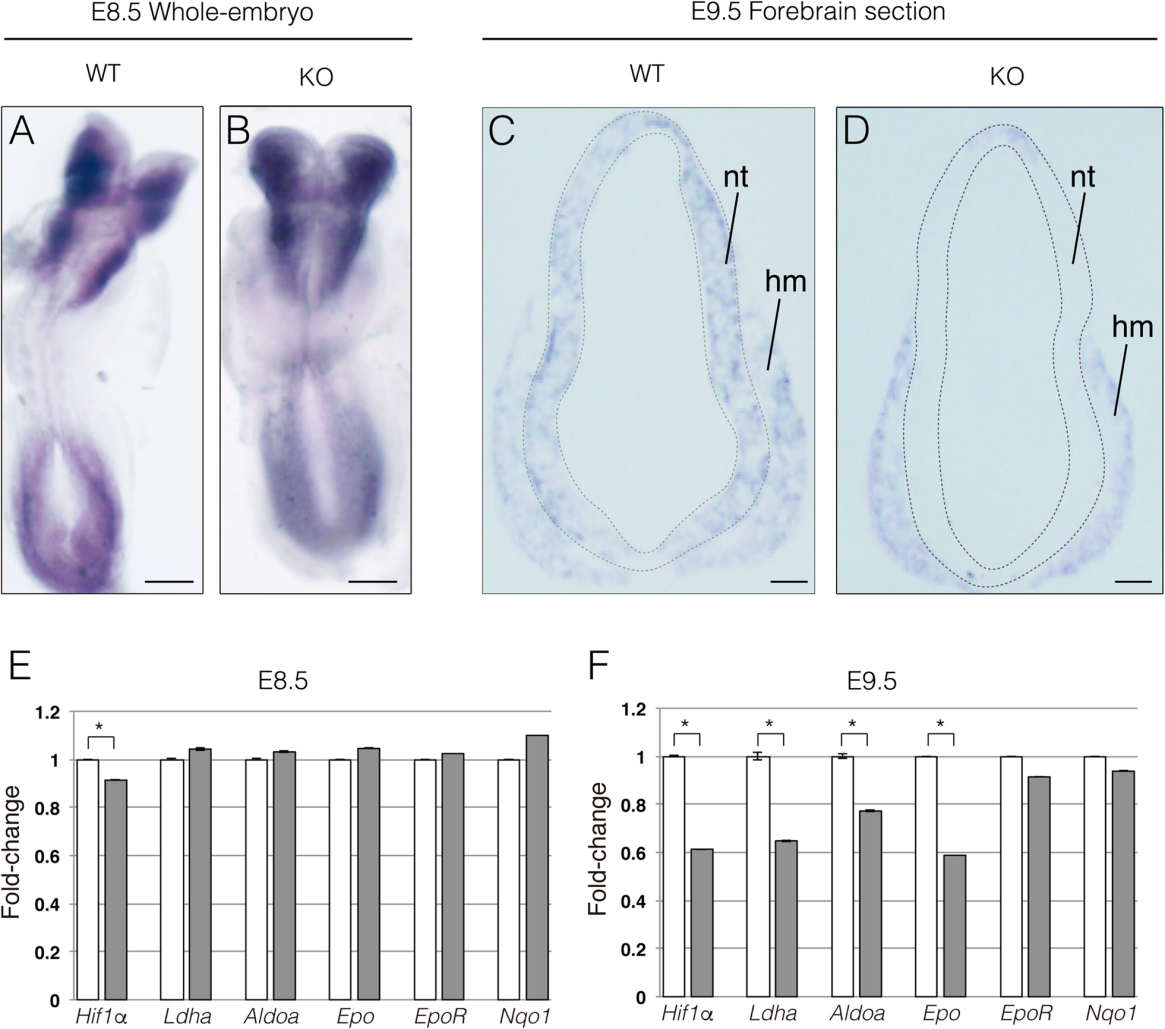
Conditional ablation of the *Hif1α* gene. Expression of *Hif1α* mRNA was detected using whole-mount and cryosection in situ hybridization of E8.5 (**A** and **B**) and E9.5 (**C** and **D**) embryos. Neural tube is indicated by the dotted line. hm, head mesenchyme; nt, neural tube; Scale bars, 100 μm. Eight (**A** and **B**) and four (**C** and **D**) independent experiments are performed and one representative image is shown, respectively. Expression levels of *Hif1α* and its target genes at E8.5 (**E**) and E9.5 (**F**) examined using RT-qPCR. The mRNA levels of target genes were normalized to those of *Gapdh*, and the relative values are presented as bars. White bar, WT; gray bar, KO. Data are the mean ± S.E.M of five embryos. Statistical differences were assessed using Student’s *t*-test, * *p* < 0.05. WT, wild type; KO, knockout

We confirmed the deletion of *Hif1α* using in situ hybridization with an antisense probe against exon 2. At E8.5, *Hif1α* mRNA was predominantly expressed in neuroepithelial cells and mesenchymal cells of WT embryos. The expression patterns and levels of *Hif1α* mRNA in KO embryos were comparable with those in WT embryos (Fig. 1A and B). At E9.5, *Hif1α* continued to be expressed in neuroepithelial cells and mesenchymal cells in WT embryos (Fig. 1C). In contrast, *Hif1α* expression diminished in the neural tube of KO embryos, although it remained unchanged in mesenchymal cells at E9.5 (Fig. 1D). To further validate the ablation of *Hif1α,* the expression of *Hif1α* and its target genes, *Ldha*, *Aldoa* (encoding a glycolytic enzyme) and *Epo* (encoding erythropoietin), was analyzed using RT-qPCR. *Hif1α* expression was slightly decreased by loss of *Hif1α*, while *Hif1α* target genes were expressed in KO embryos at levels similar to those in WT embryos at E8.5 (Fig. 1E). However, *Hif1α* expression decreased by approximately 60% in KO embryos relative to that in WT embryos at E9.5 (Fig. 1F), which was consistent with the results of in situ hybridization showing the near-complete loss of *Hif1α* transcripts in the neuroepithelium (Fig. 1D). Accordingly, the expression of Hif1α target genes was downregulated in E9.5 KO embryos (Fig. 1F; *Ldha*, reduced by 62%; *Aldoa*, by 77%; and *Epo*, by 59%). We isolated total RNA from whole mouse embryos and then prepared cDNA for RT-qPCR. Although it is preferred that total RNA is isolated exclusively from the neuroepithelium, this is technically challenging in early-stage embryos. Therefore, the reduced expression of *Hif1α* and Hif1α target genes would have been more severe if cDNA prepared from isolated neuroepithelium was used. As controls, we evaluated the expression of *EpoR* (encoding erythropoietin receptor) and *Nqo1* (encoding electron respiratory chain enzyme), which are not dependent on Hif1α. The results showed that their expression was not altered in KO embryos at E9.5 (Fig. 1F); consequently, it was inferred that the decrease in *Ldha*, *Aldoa,* and *Epo* expression levels was not due to a deficit in respiratory metabolism and erythropoiesis, but instead reflected the loss of Hif1α-dependent gene regulation in the early neuroepithelium. Taken together, these results indicate that *Hif1α* was specifically ablated in neuroepithelial cells as early as E9.5 in KO embryos.

### *Hif1α* ablation causes severe brain defects

**Fig. 2:**
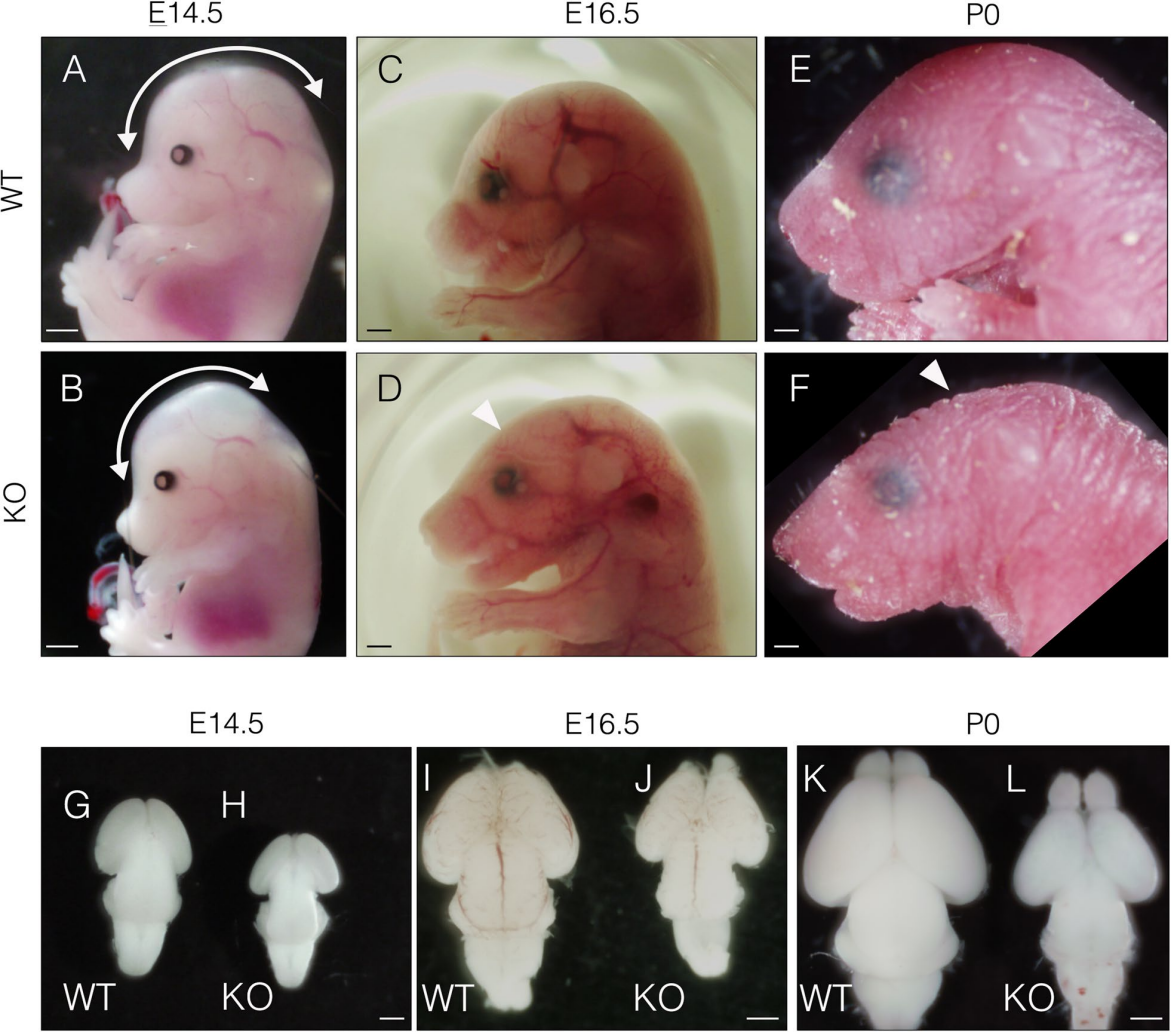
Conditional ablation of *Hif1α* in neuroepithelial cells results in a smaller telencephalon. Morphology of the head of WT (**A**, **C**, and **E**) and KO (**B**, **D**, and **F**) mice at E14.5, E16.5, and P0 are shown. Abnormally flattened parietal region is indicated by arrowheads. Scale bars, 1 mm. Morphology of whole-brain of WT (**G**, **I**, and **K**) and KO (**H**, **J**, and **L**) mice at E14.5, E16.5, and P0 are shown. Scale bars, 1 mm. WT, wild type; KO, knockout

Next, we examined the gross morphology of embryos and pups. Although KO embryos were indistinguishable from WT embryos in appearance until E12.5, they exhibited an abnormal-shaped head at E14.5. The length of the head along the sagittal axis was substantially reduced in KO embryos compared to that in WT embryos at E14.5 (Fig. 2A and B, curved two direction arrow). At E16.5, the parietal region of the head was flattened in KO embryos (Fig. 2D, arrowhead), which became more evident by P0 (Fig. 2F, arrowhead). Head abnormalities typically indicate brain hypoplasia; thus, we next examined the morphology of the brain. The brain of KO embryos was smaller than that of their WT counterparts, particularly the telencephalon at E14.5 and E16.5 (Fig. 2G–J). The brain of P0 KO pups exhibited considerable hypoplasia compared to that of WTs. Notably, the cerebrum of KO pups was extremely small compared to that of WT littermates (Fig. 2K and L). Collectively, these results demonstrate the requirement of *Hif1α* for normal development of the brain, especially that of the cerebrum.

### Neurons undergo massive apoptosis in *Hif1α*-ablated embryos

**Fig. 3:**
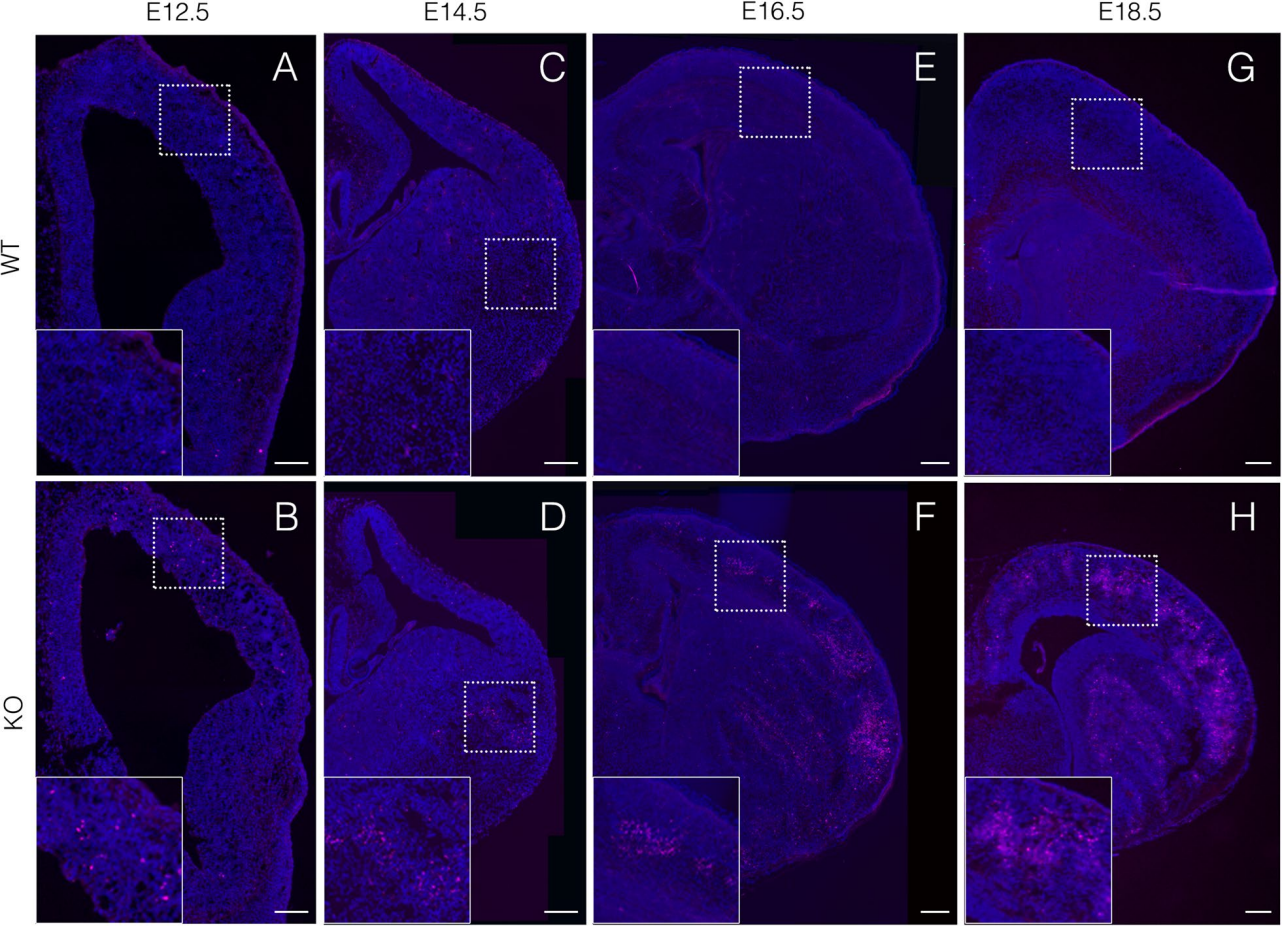
Conditional ablation of *Hif1α* in neuroepithelial cells causes massive apoptosis. Cleaved caspase3+ apoptotic cells (magenta) in coronal sections of the WT (**A**, **C**, **E**, and **G**) and KO (**B**, **D**, **F**, and **H**) telencephalon at the indicated embryonic stages are shown. Higher magnification images of the area enclosed by a rectangular dotted line are shown as insets. Scale bars, 200 μm. WT, wild type; KO, knockout. Three (**A**–**D**) and four (**E**–**H**) independent experiments are performed and one representative image is shown, respectively

Because the telencephalon was most acutely affected by the loss of *Hif1α*, we investigated the role of *Hif1α*-dependent hypoxia signaling in telencephalic development. The ablation of *Hif1α* has also been reported to induce apoptosis of neuronal cells in the telencephalon [21, 23]. Thus, we examined apoptosis using immunofluorescent labeling of cleaved caspase3, a marker of apoptotic cells. Some apoptotic cells were detected in the dorsal telencephalon of KO embryos at E12.5 (Fig. 3B). By E14.5, KO embryos displayed enhanced levels of apoptotic cells in the pallium (dorsal telencephalon) and the subpallium (ventral telencephalon) (Fig. 3D). The number of apoptotic cells in KO embryos was further increased in both the pallium and subpallium by E16.5 and E18.5 (Fig. 3F and H).

### Cerebral cortex layers are disorganized by the loss of *Hif1α*

**Fig. 4:**
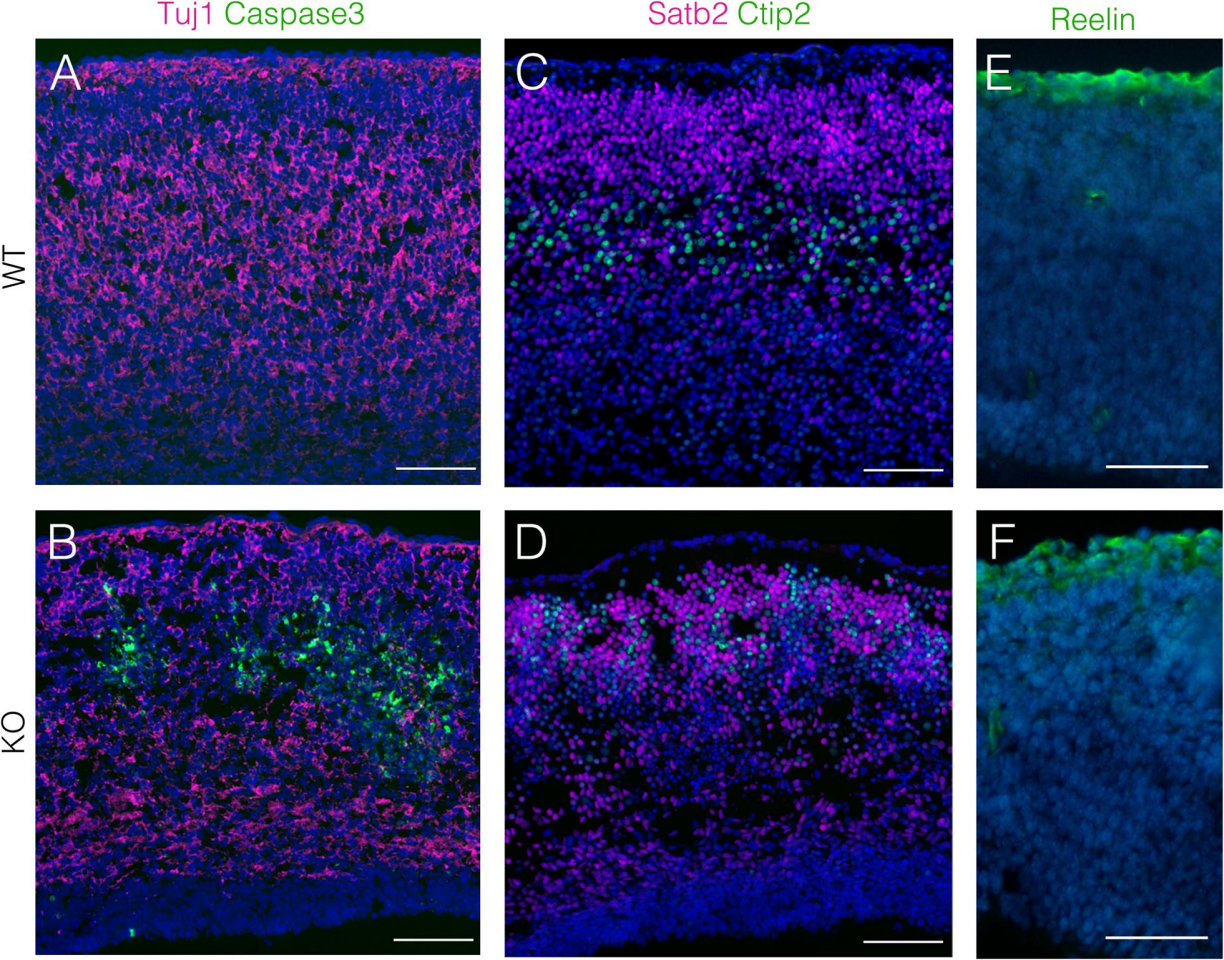
Conditional ablation of *Hif1α* in neuroepithelial cells causes neuronal apoptosis and consequently disturbs the formation of cortical layers in the cortex. Apoptotic cells and cortical neurons in coronal sections of WT and KO cortices at P0 are detected with anti-cleaved caspase3 and Tuj1 (anti-β-tubulin class III) (**A**, **B**), or anti-Satb2 and anti-Ctip2 (**C**, **D**) antibodies. Reelin expression in coronal sections of WT and KO cortices at E18.5 is detected with anti-Reelin antibody (**E**, **F**). Scale bars, 100 μm (**A**–**F**). WT, wild type; KO, knockout. Four (**A**–**D**) and three (**E** and **F**) independent experiments are performed and one representative image is shown, respectively

We further analyzed apoptosis in the KO cortex at P0. A large proportion of apoptotic cells resided in the Tuj1+ neuronal layer of the cortex, and few apoptotic cells were detected in the intermediate, subventricular, and VZ (Fig. 4B). This observation suggests that Hif1α loss led to the preferential elimination of postmitotic neurons, and consequently led to the reduction of the size of the cerebrum. We counted the number of Satb2+ and Ctip2+ cortical neurons in four arbitrarily defined regions (100 μm-wide) in the P0 cortex. Satb2 is intensely expressed in upper-layer neurons (layers II–IV), whereas Ctip2 is mainly expressed in deep-layer neurons (layers V and VI) in the cortex. The number of Satb2+ and Ctip2+ cells in the P0 cortex was reduced by approximately 88% and 49%, respectively, compared to WT controls (Table 1). This indicated that deep-layer neurons are more sensitive to *Hif1α* ablation than upper-layer neurons. Remarkably, apoptotic cells formed aggregates in the cortex (Fig. 4B), implying a disruption of the cortical layer formation in the KO cortex. The cortex consists of molecularly distinct Satb2+ upper layer and Ctip2+ deep layer projection neurons in the cerebrum of WT embryos (Fig. 4C). In contrast, each cortical layer was obscured, and Satb2+ and Ctip2+ cells were broadly scattered throughout the KO cortex (Fig. 4D).

**Table 1:** The number of Satb2- and Ctip2-positive cells in P0 cortex

	Satb2^+^ cells (%)	Ctip2^+^ cells (%)
WT	130.13 ± 8.74 (100)	32.13 ± 7.08 (100)
KO	114.25 ± 7.31 (87.80)	15.63 ± 3.08 (48.64)

The number of Satb2- and Ctip2-positive cells were counted in P0 cortex (100 μm width) on 8 histological sections obtained from 4 different pups

The disorganized cortical layers was reminiscent of the phenotype of Reelin-deficient mice, *reeler* [27]. Cajal-Retzius (C-R) cells, which occur in the most superficial marginal zone layer of the cerebral cortex, regulate cortical layer formation through the secretion of Reelin [28, 29]. Therefore, the *Sox1-Cre*-mediated deletion of *Hif1α* during early telencephalic development may have affected the survival of C-R cells. However, C-R cells were detected in the most superficial layer (layer I) of the cortex, underneath the pia, in both WT and KO telencephalons (Fig. 4E and F), suggesting that the Reelin-mediated guidance of postmitotic neurons is intact in the KO cortex.

### Neuronal migration is impaired in *Hif1α*-ablated cortex

**Fig. 5:**
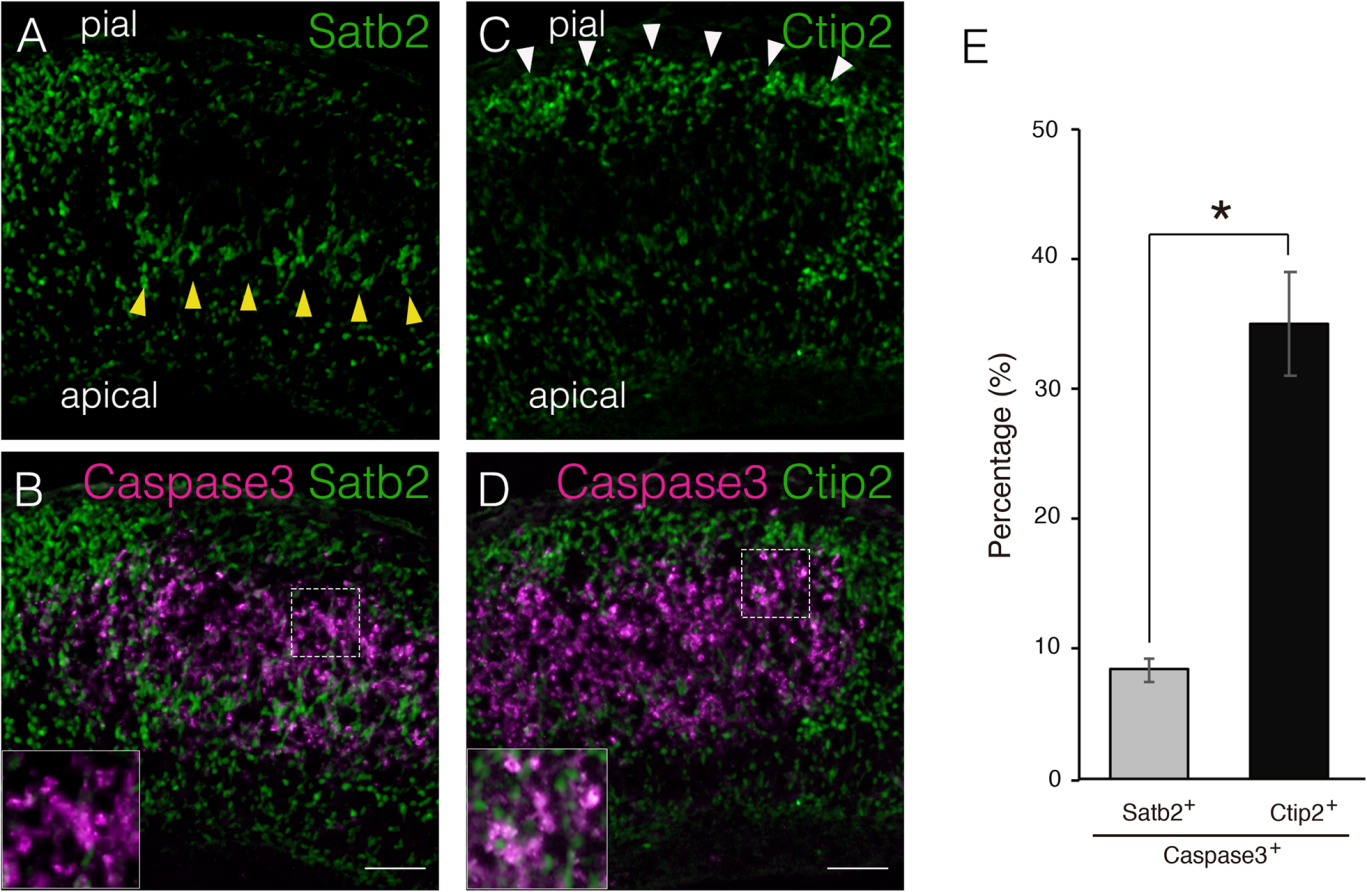
Predominant apoptotic elimination of Ctip2+ deep-layer neurons affects cortical layer formation. Apoptotic cells and Satb2+ (**A**, **B**) or Ctip2+ (**C**, **D**) cells in coronal sections of the KO cortex at E18.5 are shown. Higher magnification images of the area enclosed by rectangular dotted line are shown as insets (**B**, **D**). Satb2+ cells accumulating at the apical side of the apoptotic cell aggregate are indicated by yellow arrowheads. Ctip2+ cells distributed near the pial surface are indicated by white arrowheads. Scale bars, 100 μm (**A**–**D**). Three independent experiments are performed and one representative image is shown, respectively. The percentage of cleaved caspase3 + and Satb2+ or Ctip2+ cells is presented as a bar graph (**E**). Data are the mean ± S.E.M of six sections. Statistical differences were assessed using Student’s *t*-test, * *p* < 0.05

To explore the mechanism inducing neuronal apoptosis by loss of *Hif1α*, we examined whether upper- or deep-layer neurons undergo apoptosis via immunofluorescent staining using anti-cleaved caspase3 and anti-Satb2 or anti-Ctip2 antibodies (Fig. 5A–D). We observed that many Ctip2+ cells were co-labeled with cleaved caspase3 (Fig. 5D, inset). The number of Satb2+ /cleaved caspase3+ double-labelled cells was lower than that of Ctip2+ /cleaved caspase3+ cells in the E18.5 KO cortex (Fig. 5B, inset). Specifically, approximately 8.4% and 35.1% of total cleaved caspase3+ cells were co-labeled with Satb2 and Ctip2, respectively (Fig. 5E). This demonstrated that deep-layer neurons preferentially undergo apoptosis in the absence of *Hif1α*, which was consistent with data showing a predominant reduction of deep-layer neurons in the KO cortex (Table 1). Approximately 56% of all cells were cleaved caspase3+ cells, thereby explaining the severe loss of layer marker expression.

**Fig. 6:**
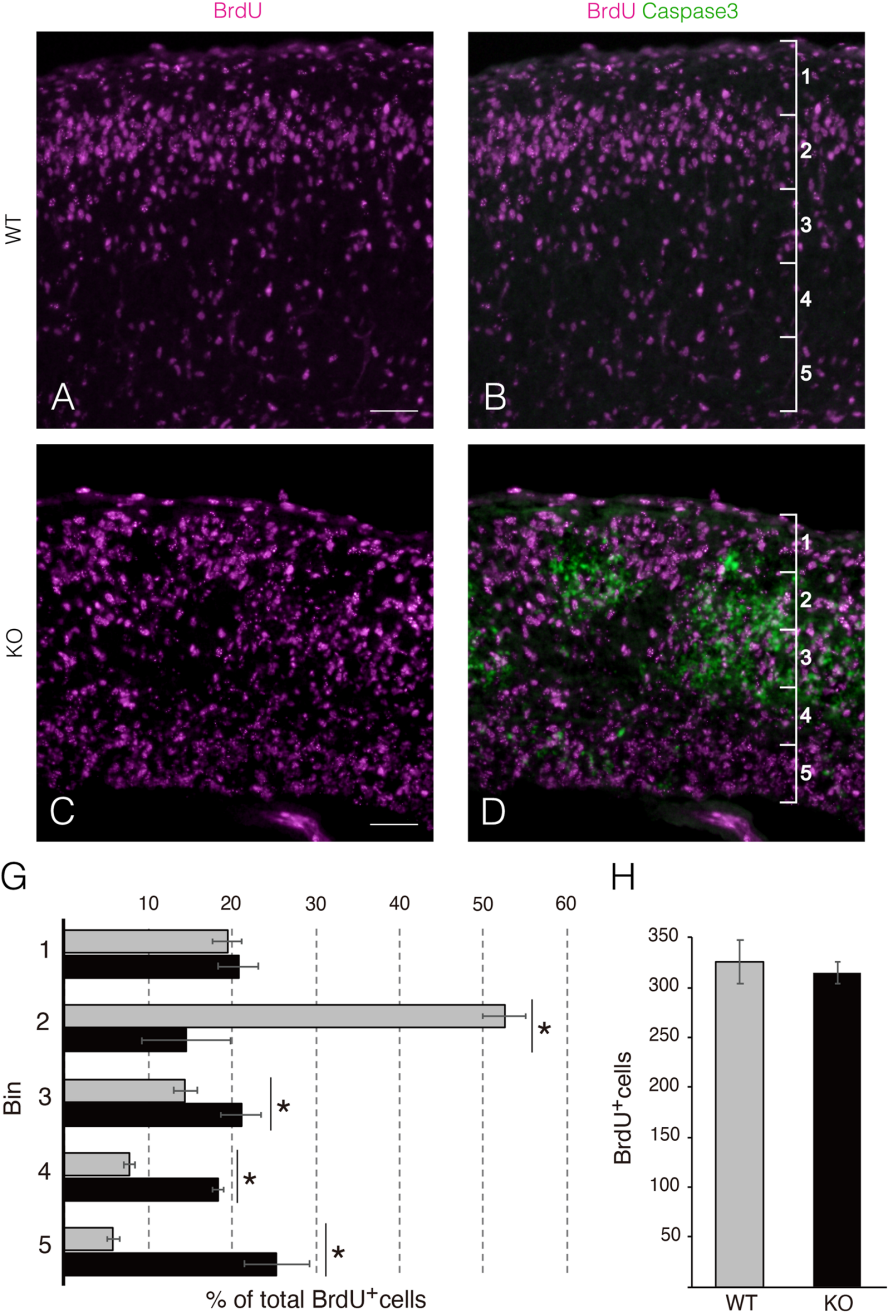
Neural migration of upper-layer neurons is impaired by loss of *Hif1α*. Apoptotic cells and BrdU-incorporated neurons in coronal sections of WT and KO cortices at E18.5 are detected with anti-cleaved caspase3 and anti-BrdU antibodies (**A**–**D**). Scale bars, 100 μm (**A**–**D**). Three independent experiments are performed and one representative image is shown, respectively. The percentage of BrdU+ cells in the cortex (bins 1–5, 500 μm-wide) is presented as a histogram (**G**). The total number of BrdU+ cells in the cortex (500 μm-wide region) is presented as a bar graph (**H**). Gray bar, WT; black bar, KO. Data are the mean ± S.E.M of four sections. Statistical differences were assessed using Student’s *t*-test, * *p* < 0.05. WT, wild type; KO, knockout

Many Satb2+ upper-layer neurons were detected around apoptotic cell aggregates, especially on the apical side of the KO cortex (Fig. 5A, yellow arrowheads). Consistent with this, Satb2+ cells were sparsely distributed on the pial surface side of apoptotic cell aggregates. Conversely, Ctip2+ neurons were abnormally detected near the pial surface side where the upper layers were formed in the normal cortex (Fig. 5C, white arrowheads). The cerebral cortex consists of six neuronal layers that develop in an inside-out manner, i.e., early-born neurons settle in the deep layers, whereas late-born neurons migrate through the deep-layer neurons and form more superficial layers (upper-layers) [30]. Thus, we hypothesized that the migration of the upper-layer neurons from the VZ to the pial surface was impeded by apoptosis of deep-layer neurons, resulting in a partial reversal of the cortical layer in the KO cortex. To test this hypothesis, we performed a BrdU birthdating assay. To label the upper-layer neurons, BrdU was injected into pregnant mice at E14.5, when upper-layer neurons are being born, and then the position of BrdU-labeled neurons were compared between KO and WT cortices at E18.5. A large proportion of BrdU+ neurons were located in the upper-layers of the WT cortex (corresponding to bins 1 and 2; Fig. 6A and B). In contrast, many BrdU+ neurons were detected in the deeper-layers of the KO cortex. Importantly, most BrdU+ neurons were negative for apoptosis markers (Fig. 6C and D), which is consistent with the results shown in Fig. 5B and E. The distribution of BrdU+ neurons born at E14.5 was quantified in five arbitrarily defined regions (500 μm-wide) in the cortex. In the WT cortex, approximately 72% of BrdU+ neurons were present in the upper-layers (bins 1 and 2). In contrast, the percentage of BrdU+ neurons in the presumptive area of the upper-layers (bins 1 and 2) was reduced to approximately 36% in the KO cortex (Fig. 6G). Approximately 42% of BrdU+ neurons populated the middle area (bins 3 and 4) in the KO cortex, where a large number of apoptotic cells were observed. In contrast, approximately 21% of BrdU+ neurons were detected in the same region of the WT cortex (Fig. 6G). Notably, the total number of BrdU+ neurons in the KO cortex was comparable with that of BrdU+ neurons in the WT cortex, suggesting that the proliferation of neural progenitors of upper-layer neurons was not altered by the loss of *Hif1α* (Fig. 6H). Taken together, these results strongly support our hypothesis that the migration of upper-layer neurons was impeded by massive apoptosis of deep-layer neurons in the KO cortex.

Postmitotic neurons migrate towards the pia along the radial glial cell fiber spanning the length of the developing cortical plate, and is important for proper ‘inside-out’ neural migration [27, 28, 30]. Almost radial glial fibers spanned the entire cortex in both of WT and KO telencephalon (Additional file 2: Figure S2). This result suggests that the radial glial fibers-dependent neural migration is intact in the KO cortex.

### Possible mechanism underlying the role of Hif1α in cortical development

**Fig. 7:**
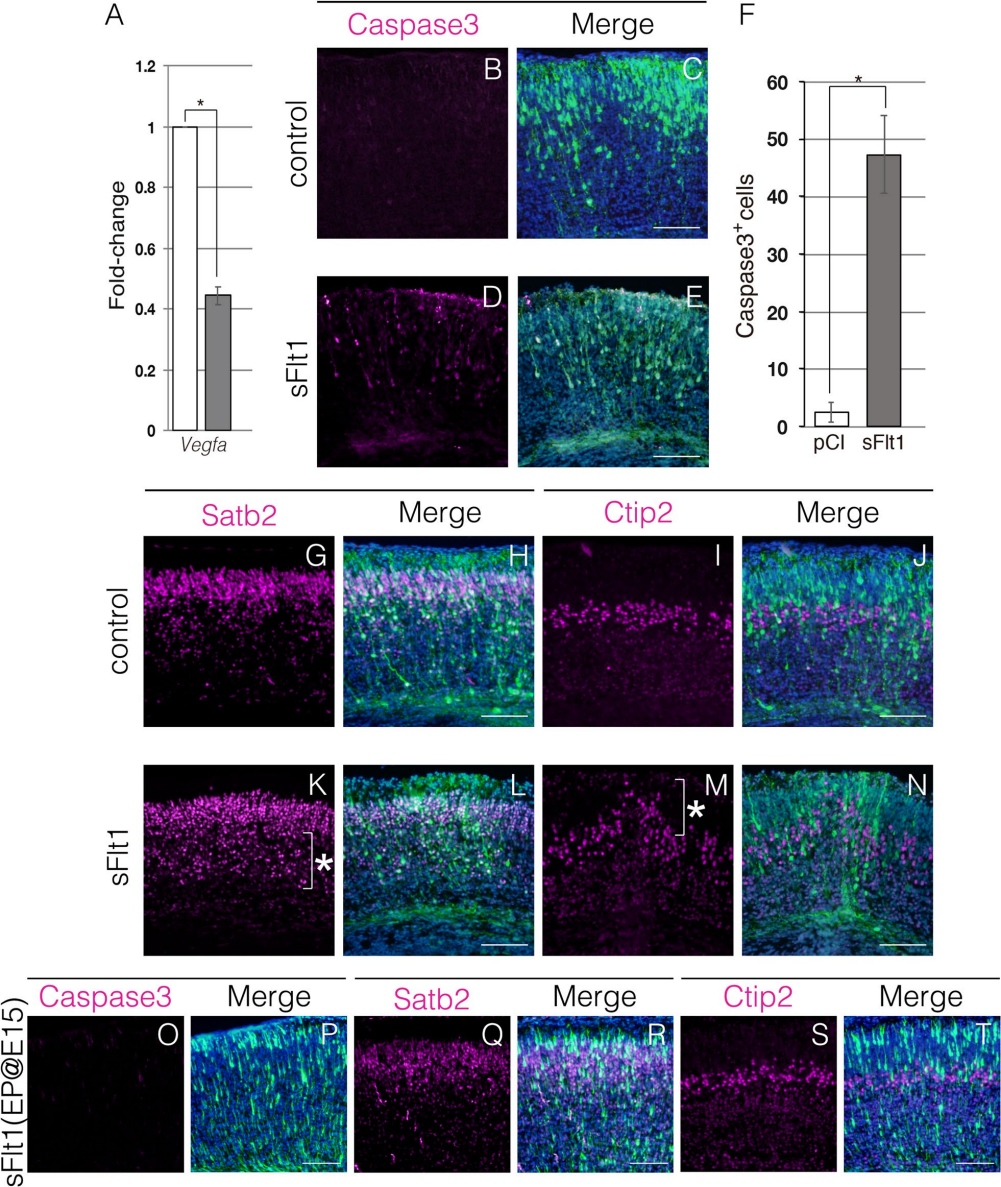
Vegf signaling is required for the survival of deep-layer neurons and proper cortex formation. *Vegf* expression level was quantified using RT-qPCR (**A**). The mRNA levels of *Vegf* mRNA normalized to that of *Gapdh*. The relative values are presented as a bar graph. White bar, WT; gray bar, KO. Data are the mean ± S.E.M of 3 embryos. Statistical differences were assessed using Student’s *t*-test, * *p* < 0.05. pCI (control) or sFlt1-pCI (sFlt1) plasmid was electroporated into neural progenitor cells at E13.5 (**B**–**E**, **G**–**N**). Apoptotic cells (magenta) and transfected cells (green) are detected with anti-cleaved caspase3 and anti-EGFP antibodies, respectively, in coronal sections of the cortex at E18.5 (**B**–**E**). The total number of cleaved caspase3+ cells in the cortex (500 μm-wide region) is presented as a bar graph (**F**). White bar, control; gray bar, sFlt1. Data are the mean ± S.E.M of five sections. Statistical differences were assessed using Student’s *t*-test, * *p* < 0.05. Indicated cortical neurons (magenta) and transfected cells (green) are detected with anti-Satb2 (**G**, **H**, **K**, **L**) or anti-Ctip2 (**I**, **J**, **M**, **N**) and anti-EGFP antibodies, respectively, in coronal sections of the cortex at E18.5. Abnormal localization of cortical neurons is indicated with the asterisk. sFlt1-pCI plasmid was electroporated into neural progenitor cells at E15.5 (**O**–**T**). Apoptotic cells (magenta) and transfected cells (green) with anti-cleaved caspase3 and anti-EGFP antibodies, respectively, in coronal sections of the cortex at E18.5 (**O** and **P**). Indicated cortical neurons (magenta) and transfected cells (green) with anti-Satb2 (**Q** and **R**) or anti-Ctip2 (**S** and **T**) and anti-EGFP antibodies, respectively, in coronal sections of the cortex at E18.5. Scale bars, 100 μm (**B**–**E**, **G**–**T**). Three (**B**–**E**, **O**–**T**) and four (**F**–**M**) independent experiments are performed and one representative image is shown, respectively

Previous studies demonstrated that vascular endothelial growth factor (encoded by *Vegf)*, a downstream target of *Hif1α*, functions not only as an angiogenic growth factor but also as a neurotrophic factor [31, 32]. It has also been demonstrated that neuronal cell-specific *Vegf* homozygous knockout mice die within 24 h after birth due to the dysmorphogenesis of the cortex caused by massive neuronal apoptosis [33, 34]. Moreover, as apoptotic cells seem to accumulate in the middle area of the cortex [33], the *Vegf* homozygous knockout phenotype resembles that of our *Hif1α* homozygous knockout mice. We therefore investigated if the reduced *Vegf* expression was associated with reduced survival of deep-layer neurons upon *Hif1α* loss. *Vegf* is expressed in the ventral layer of the brain at E14.5 [35]. First, we analyzed *Vegf* expression levels in WT and KO telencephalons at E13.5, when deep-layer neurons are born. To analyze this, the dorsal part of the telencephalon, including the cortex, was surgically isolated, and RNA was prepared for RT-qPCR. We confirmed a significant reduction in *Vegf* expression in the cortex of KO mice (Fig. 7A). Next, we examined whether Vegf signaling activity is required for the survival of deep-layer neurons. Although the suppression of Vegf receptor function is the ideal method to inhibit the input of Vegf signaling, known receptors for Vegf, such as Flk1, Flt1, Nrp1, and Nrp2, do not appear to be involved in cortical development and thus unlikely targets to manipulate Vegf signaling in the developing brain [34, 36–38]. Therefore, we took advantage of the ligand-binding domain of the Vegf receptor, sFlt1, to interfere with Vegf signaling [39]. It has been reported that the overexpression of sFlt1 inhibits Vegf signaling in vivo [40]*.* We thus hypothesized that secreted sFlt1 functions as a decoy Vegf receptor and inhibits paracrine and/or autocrine Vegf signaling inputs to neural progenitors and/or neurons of deep layers. The expression construct of mouse sFlt1 was introduced into neural progenitor cells of E13.5 WT embryos using in utero electroporation along with the EGFP expression construct. At E18.5, numerous EGFP + sFlt1-overexpressing cells were co-labeled with apoptotic markers, while no apoptosis was observed in the control cortex (Fig. 7B–F). However, we could not detect apoptotic cell aggregates, which may be due to the variation in transfection efficiency of sFlt1 (Fig. 7D and E). Next, we investigated cortical layer formation in the sFlt1-transfected cortex. Satb2+ neurons were dispersed in the apical side of the cortex where deep-layer neurons normally settle in the control cortex (Fig. 7G, H, K, L). The Ctip2+ neuron layer was considerably shifted to the pial surface in the transfected region of sFlt1, consistent with the accumulation of upper-layer neurons in the deep layers of the KO cortex (Fig. 7I, J, M, N). In contrast, apoptosis and cortical layer defects were not induced when the sFlt1-expressing construct was electroporated into neural progenitor cells at E15.5 (Fig. 7O–T), suggesting that Vegf signaling is not required for the survival of upper-layer neurons. Taken together, inhibition of Vegf signaling by the overexpression of sFlt1 mimics cortical development defects seen in KO embryos.

**Fig. 8:**
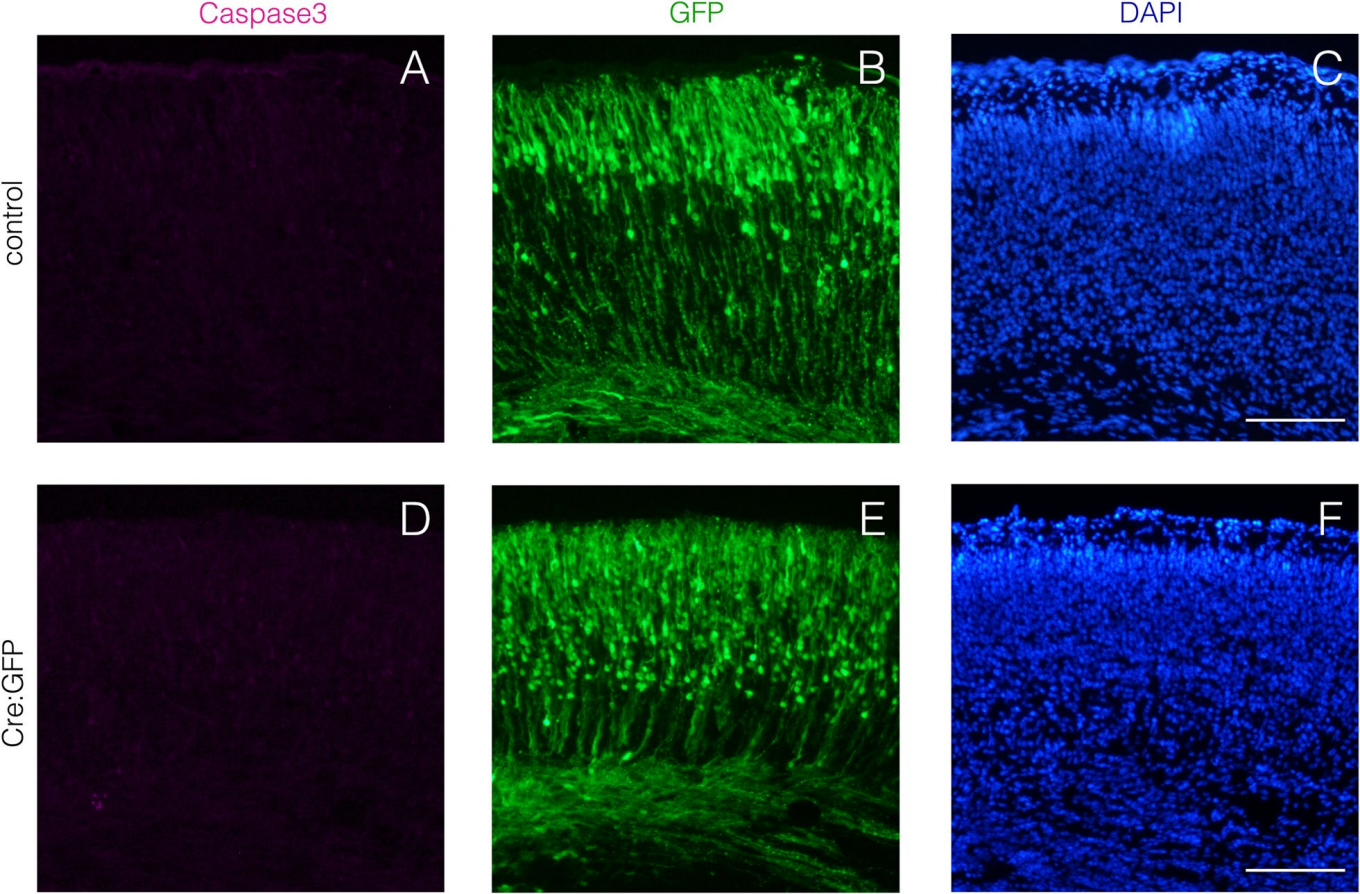
Vegf functions as the survival factor for deep-layer neurons in non-cell-autonomous manner. pEGFP-CAGS (control) or pCAG-Cre:GFP (Cre:GFP) plasmid was electroporated into neural progenitor cells in one side of telencephalic semisphere of *Hif1α *^*flox/flox*^ embryo at E13.5. Cleaved caspase3+ apoptotic cells (magenta) and EGFP+ transfected cells (green) were detected in coronal sections of the cortex at E18.5. Scale bars; 100 μm. Three independent experiments are performed and one representative image is shown, respectively

As we postulated that neuronal apoptosis is due to the decrease in Vegf levels in the KO cortex, it is important to identify the source of Vegf in the telencephalon. Unfortunately, we could not detect the expression of *Vegf* mRNA and Vegf protein in the telencephalon using in situ hybridization and immunofluorescence (data not shown). Instead of that, we tried to determine whether Vegf functions in cell-autonomous or non-cell-autonomous manner. The expression construct of Cre recombinase was introduced into E13.5 *Hif1α *^*flox/flox*^ telencephalon using in utero electroporation. At E18.5, EGFP + *Hif1α*-ablated cells did not undergo apoptosis (Fig. 8A), suggesting the non-cell-autonomous function of Vegf for the survival of deep-layer neurons. Notable, many EGFP + *Hif1α*-ablated cells were localized more apical side compared to EGFP+ cells in control cortex (Fig. 8B and E). This result indicates that, besides regulating the survival of deep-layer neurons in non-cell-autonomous manner, *Hif1α* functions cell-autonomously in neuronal migration.

## Discussion

**Fig. 9:**
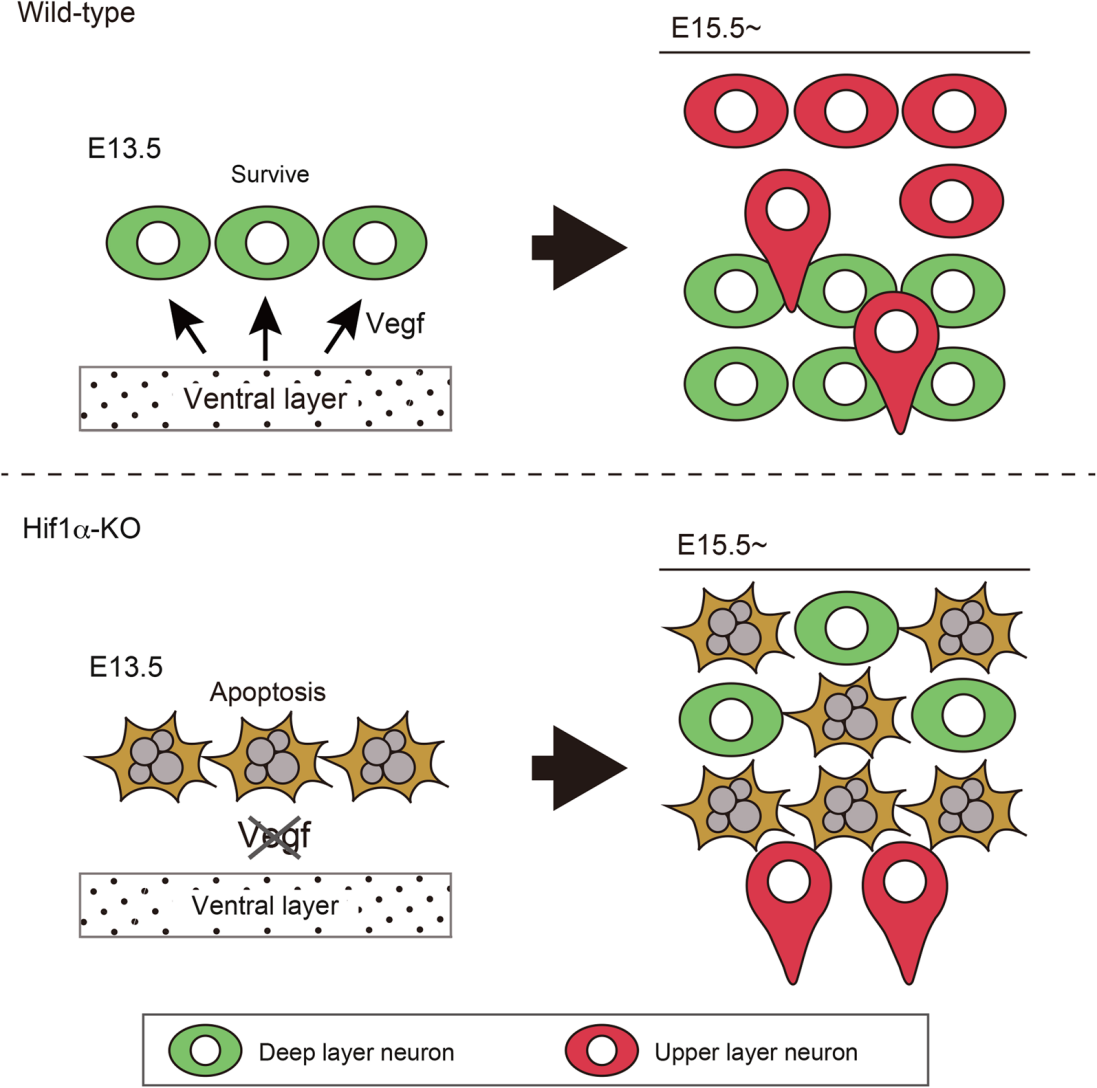
Summary of *Hif1α* function in the development of telencephalon. Vegf is expressed in a Hif1α-dependent manner and acts as a survival factor for deep-layer neurons. Apoptotic elimination of deep-layer neurons affects the migration of upper-layer neurons passing deep layers

In the present study, we elucidated the role of *Hif1α* in early-stage telencephalic development. We observed that neuroepithelial cell-specific ablation of *Hif1α* causes massive apoptosis of neurons in the telencephalon and cerebral cortex, which manifests as reduced brain size. We demonstrated that deep-layer neurons predominantly undergo apoptosis by *Hif1α*-ablation, and their loss affects the radial migration of upper-layer neurons and subsequent cortical layer formation. Furthermore, we showed that Vegf acts as a neurotrophic factor likely acting downstream of Hif1α to promote the survival of deep-layer neurons (Fig. 9).

Neural cell-specific knockout of *Hif1α* using *Nestin-Cre* driver causes massive apoptosis in the cerebrum at E19, resulting in hydrocephalus at P70, while no gross morphological differences can be observed in E15 embryos [23]. In our KO embryos, apoptosis occurred as early as E12.5, and gross morphological abnormalities of the head were apparent at E14.5 (Fig. 2). These phenotypic differences are presumably the result of the timing of Cre-mediated recombination in the *Nestin-Cre* vs. *Sox1-Cre* driver alleles. In the *Nestin-Cre* mouse line, *Cre* mRNA expression is detected by E11.5; however, it is unclear if Cre is expressed at earlier stages [24]. In contrast, Cre-mediated recombination was evident as early as E9.5 in our *Sox1-Cre*-mediated *Hif1α* KO embryos (Fig. 1D and F). Liang et al*.* indicated that Cre-mediated recombination occurs at sufficient levels in postmitotic neuronal cells, and not only in neural progenitors in *Nestin-Cre* embryos [25]. Thus, by restricting the recombination to early neuroepithelial cells using the *Sox1-Cre* driver, we produced a more complete neural-specific ablation of *Hif1α* and revealed a specific function for the gene in early telencephalic development. *Sox1* mRNA expression begins at the anterior neuroepithelium of wild-type mouse embryos at E8.0 [41]. Consistent with this, Cre recombination activity has been detected in the neuroepithelium of *Sox1-Cre; ROSA26R-EYFP*, at least E8.5 onward [26]. However, Cre-mediated recombination was not detected at E8.5 in *Sox1-Cre*-mediated *Hif1α* KO embryos using in situ hybridization. The delay in Cre activity may be related to the difference in recombination efficiency of the *ROSA26R* allele compared to that of the *Hif1α *^*flox/flox*^ allele. Dorsal forebrain radial glial cells-specific knockout of *Hif1α* using *Emx1-Cre* driver also causes apoptosis in the telencephalon. *Emx1-Cre*-mediated homozygous *Hif1α* mutant mice are viable and fertile, similar with *Nestin-Cre*-mediated homozygous *Hif1α* mutant mice. Notably, heterozygous ablation of *Hif1α* using *Emx1-Cre* driver causes precocious neurogenic differentiation of neural progenitor cells, resulting in mild microcephaly [21]. In contrast, heterozygous ablation of *Hif1α* using *Sox1-Cre* driver did not alter the number of Sox2+ apical neural progenitor cells and Tbr2+ basal neural progenitor cells (Additional file 3: Figure S3). We thus speculate that these phenotypic differences are due to the timing, or the level of Cre expression in the *Emx1-Cre* vs. *Sox1-Cre* driver alleles.

Massive apoptosis of deep-layer neurons appears to be the major cause of cortical layer formation deficiency in the KO cortex. However, the mechanism by which apoptotic elimination disturbs the migration of upper-layer neurons is still elusive. One possibility is the loss of the chemoattractant gradient for upper-layer neurons toward the pia. For example, the endogenous gradient of Semaphorin-3A (*Sema3A*) functions as a guidance factor and regulates the migration of upper-layer neurons [42]. *Sema3A* mRNA is highly expressed in outer cortical layer neurons of the rat cortex from E14 (nearly equal to E12 mouse cortex) which correspond with deep-layer neurons. Thereafter, Sema3A protein is detected across the rat cortex in a graded manner from E18 (equivalent to the E16 mouse cortex). The gradient distribution of Sema3A may be not formed by apoptotic elimination of deep-layer neurons in KO cortex; consequently, upper-layer neurons may not be able to pass through the deep layers. Another possibility is the disruption of heterogeneous cell–cell interactions of migrating neurons. Del Toro et al. have reported that Flrt1and 3, cell adhesion molecules expressing in cortical neurons regulate the migration of neurons through repulsive interactions with surrounding neurons [43]. Flrt1/3 expressing upper-layer neurons may lose the interaction partner due to the apoptosis, and display abnormal migration in KO telencephalon. Furthermore, apoptotic cell aggregates may physically block the migration path of upper-layer neurons in the KO brains. The molecular mechanism of neuronal migration defects caused by the loss of *Hif1α* remains to be elucidated, and further experiments are needed to shed light on this*.*

Transfection of Cre-expression construct into *Hif1α *^*flox/flox*^ neural progenitor cells did not induce apopotosis in the cortex. This finding strongly suggested non-cell-autonomous function of Vegf for deep-layer neuron survival. In *Vegf*-LacZ knock-in mice, *Vegf* expression was detected in the ventral layer of the brain at E14.5 [35]. Based on these findings, we assumed that Vegf is primarily secreted from neural progenitor cells in the ventricular zone and that it probably acts as a paracrine neurotrophic factor to postmitotic or mature deep-layer neurons (Fig. 9). Although certain unresolved points remain; it is unknown how Vegf signaling is activated specifically in deep-layer neurons and which receptor(s) mediate Vegf signaling. Considerable efforts are required to resolve these issues.

There lacks a consensus of whether Vegf directly regulates neuronal survival as a neurotrophic factor or indirectly maintains neurons through vascular formation. The telencephalon of KO mice exhibited the decrease in the density of the vessels (Additional file 4: Figure S4). In the KO telencephalon, blood vessels regressed and formed punctate structures in the proximity of apoptotic cell aggregates, instead of elongated networks (Additional file 4: Figure S4F, arrowheads). A similar phenotype is observed in the *Nestin-Cre*-mediated *Hif1α* KO embryos, suggesting that a deficiency of vascular network formation impacted on the survival of neurons [23]. Therefore, it is difficult at present to define the cellular mechanism of Vegf signaling in deep-layer neuron survival. However, our overexpression experiment showed that vascular networks normally develop whereas cortical layer formation is disturbed in the sFlt1-transfected cortex (Additional file 5: Figure S5). These data suggest the primary role of Vegf as a survival factor for neurons rather than an endothelial cell growth factor during cortical development. In support of our findings, Lange et al*.* demonstrated that Gpr124-LacZ knock-in null embryos do not exhibit any neuronal apoptosis, even though the vasculature is largely diminished in the cortex [21]. In addition, the formation of the cortical vascular network is non-cell autonomously regulated by Vegf, which is released from neurons [36]. Collectively we speculate that Vegf expression might reduce by neuronal apoptosis, and the deficiency in Vegf affect vascular outgrowth and extension in cortical tissue of KO brain. Our findings emphasize that the function of Vegf for the survival of neurons is essential for cortical layer formation. However, we can not exclude the possibility that Vegf regulates neural migration as well. In future studies it will be important to confirm this possibility.

In conclusion, our findings provide insights into the molecular mechanisms underlying the development of murine embryonic telencephalon, which is regulated by *Hif1α*-dependent hypoxia signaling.

## Methods

### Mice

All mice were maintained on a C57BL/6 background. *Hif1α *^*flox/flox*^ mice (Stock No. 007561) and *ROSA26/CAG-floxed STOP-tdTomato* mice (Stock No. 007905) were obtained from The Jackson Laboratory, and *Sox1-Cre*^+/−^ mice from the RIKEN Bioresource Research Center (Accession No. CDB0525K, http://www2.clst.riken.jp/arg/mutant%20mice%20list.html) [26]. *Hif1α *^*flox/flox*^ mice and heterozygotes (*Hif1α *^*flox/wt*^; *Sox1-Cre*^+/−^) were crossed to obtain homozygotes (*Hif1α *^*flox/flox*^; *Sox1-Cre*^+/−^). For embryonic staging, the morning on which the vaginal plug was observed was designated as E0.5.

### RT-qPCR

Total RNA was extracted from E8.5 and E9.5 WT and KO embryos, or the cortex of E13.5 WT and KO brains using the RNeasy Mini Kit (Qiagen) according to the manufacturer’s instructions. Isolated RNA was then reverse-transcribed to cDNA using PrimeScript 1st strand cDNA Synthesis Kit (Takara) according to the manufacturer’s instructions. qPCR using SYBR green was performed on a LightCycler Nano System (Roche). Gene expression was normalized to *Gapdh* expression levels. Each sample was analyzed at least in triplicate. The relative fold change was calculated using the 2^−ΔΔCt^ method. For detection of *Hif1α*, *Ldha, Aldoa, Epo, EpoR, Nqo1, Vegfa*, and *Gapdh* expression levels, the following primers were used: *Hif1α* FW: TGAGCTTGCTCATCAGTTGC, *Hif1α* RV: CATAACAGAAGCTTTATCAAGATGTGA; *Ldha* FW: GGCACTGACGCAGACAAG, *Ldha* RV: TGATCACCTCGTAGGCACTG; *Aldoa* FW: TGGGAAGAAGGAGAACCTGA, *Aldoa* RV: GACAAGCGAGGCTGTTGG; *Epo* FW: TCTGCGACAGTCGAGTTCTG, *Epo* RV: CTTCtGCACAACCCATCGT; *EpoR* FW: GTCCTCATCTCGCTGTTGCT, *EpoR* RV: ATGCCAGGCCAGATCTTCT; *Nqo1* FW: AGCGTTCGGTATTACGATCC, *Nqo1* RV: AGTACAATCAGGGCTCTTCTCG; *Vegfa* FW: CTTGTTCAGAGCGGAGAAAGC, *Vegfa* RV: ACATCTGCAAGTACGTTCGTT; *Gapdh* FW: CATGTTCCAGTATGACTCCACTC, *Gapdh* RV: GGCCTCACCCCATTTGATGT.

### In situ hybridization

A part of the mouse *Hif1α* sequence was PCR-amplified using the following primer set: FW-*Hif1α*: AAAGAGTCTGAAGTTTTTTATGAG and RV-*Hif1α*: CTTCTTAAGCTTATCAAAAAGGCA. The PCR products were cloned into the pGEM-T Easy vector (Promega) to synthesize the cRNA probe.

Whole-mount and cryosection in situ hybridization was performed as described previously [44, 45].

### Immunofluorescence

Mouse brains were fixed with 4% paraformaldehyde (PFA) in phosphate-buffered saline (PBS) for 3 h at 4 ℃ and cryopreserved in 30% sucrose in PBS. Brains were embedded in optimal cutting temperature compound (OCT) and stored at − 80 ℃ until further use. The cryostat sections were cut at 10 μm and adhered onto glass slides. The sections were washed with PBS and then incubated with 10 mM citric acid (pH 6.0) for 30 min at 80 ℃ for antigen retrieval. After washing with PBS briefly, sections were incubated with 0.5% Triton X-100 in PBS for 15 min at room temperature and then with blocking buffer (3% BSA in TBST) for 30 min at room temperature. Further, the sections were incubated at 4 °C overnight with primary antibodies against Nestin (RC2, DSHB; 1/100), Sox2 (AF2018, R&D sysytems; 1/200), Tbr2 (ab23345, Abcam; 1/1000), cleaved caspase3 (9661, Cell Signaling; 1/200), Reelin (MAB5364, Chemicon; 1/1000), β-tubulin class III (Tuj1, PRB-435P, Covance; 1/1000), Satb2 (ab51502, Abcam; 1/500), and Ctip2 (ab18465, Abcam; 1/500). To visualize the vasculature, IB4-FITC (Sigma, 1/100) was used. Sections were washed thrice with TBST for 10 min and then incubated with the appropriate secondary antibodies conjugated with Alexa 488 or 546 (A11001, A21208, Invitrogen; 1/300) for 1 h at room temperature. Nuclei were stained with DAPI. Fluorescence microscopy was performed on a BX51 microscope equipped with a DP30BW CCD camera (Olympus) using 10× and 20× objective lenses. Images were acquired using the DP controller software (Olympus).

### BrdU birthdating assay

Pregnant mice (E14.5) were injected intraperitoneally with BrdU at 0.1 mg/kg of body weight and euthanized at E18.5. Brains were fixed with 4% PFA solution in PBS for 3 h at 4 ℃ and cryopreserved in 30% sucrose in PBS. Brains were embedded in OCT and stored at − 80 ℃ until further use. The cryostat sections were cut at 12 μm and adhered onto glass slides. Then, the sections were washed with PBS and incubated with 10 mM citric acid (pH 6.0) for 30 min at 80 °C for antigen retrieval. The sections were incubated with 0.1 N HCl for 1 h at 37 °C. After incubation and brief washing with PBS, the sections were incubated with 0.5% Triton X-100 in PBS for 15 min at room temperature, and then incubated with blocking buffer (10% horse serum in TBST) for 30 min at room temperature. Further, the sections were incubated at 4 °C overnight with primary antibodies against BrdU (347,580, BD Bioscience; 1/100) and cleaved caspase3 (9661, Cell Signaling; 1/200), washed twice with TBST for 10 min, and incubated with the appropriate secondary antibodies conjugated with Alexa 488 or 546 (A11001, A21208, Invitrogen; 1/300) for 1 h at room temperature. Fluorescence microscopy was performed on a BX51 microscope equipped with a DP30BW CCD camera (Olympus) using 10× and 20× objective lenses. Images were processed using the DP controller software (Olympus).

### In utero electroporation

In utero electroporation was performed as previously described, with slight modifications [46]. Briefly, pregnant mice at E13.5 or E15.5 were anesthetized by intraperitoneal (IP) injection of saline containing butorphanol (5 mg/kg body weight), midazolam (4 mg/kg body weight), and medetomidine (0.3 mg per kg body weight), and then the uterine horns were exposed. Approximately 1 μL of plasmid DNA mixture with 0.01% Fast Green solution was injected into the lateral ventricle of embryos using a fine-tip glass micropipette (G-1.0, Narishige). The final concentration of plasmid DNA was as follows: pEGFP-CAGS (0.5 or 1 μg/μL), m-sFlt1-pCI (1 μg/μL), pCI (1 μg/μL) and pCAG-Cre:GFP (1 μg/μL). Electric pulses (50 V, 1 pulse and 35 V, 4 pulses; 50 ms on, 950 ms off) were delivered to the dorsomedial part of the cortex using a forceps-type electrode (5 mm diameter, BEX) through CUY21 EDIT II (BEX). After wound closure, anesthesia was terminated by IP injection of atipamezole (3 mg/kg body weight). The embryos were then allowed to develop to E18.5.

### Statistics

For statistical analysis, two-tailed Student's *t*-test was used to determine *p*-values, and values with *p* less than 0.05 were considered statistically significant.

## Supplementary Information

**Additional file 1: Figure S1.** Cre is activated in neural progenitor cells and neural cells. IB4-FITC-positive blood vessels (green), tdTomato-positive cells (magenta), and nucleus (blue) were detected in coronal sections of *Sox1-Cre*^+/−^*; ROSA26/CAG-floxed STOP-tdTomato*^+/−^ telencephalon at E16.5. Conforcal optical slices were collected and maximum-intensity projections of 15 μm stacks were made. Scale bars; 10 μm. Three independent experiments are performed and one representative image is shown, respectively.

**Additional file 2: Figure S2.** Radial glial fibers are normally formed in KO telencephaon. Morphology of radial glial cell fibers were analyzed by immunofluorescence using anti-Nestin antibody in coronal sections of WT (A and B) and KO (C and D) telencephalon at E13.0. B and D are magnifications of the region enclosed with dotted-line in A and C, respectively. White arrowheads indicate a radial glial fiber. Scale bars; 100 μm. Five independent experiments are performed and one representative image is shown, respectively.

**Additional file 3: Figure S3.** Heterozygous *Hif1α* ablation does not affect the development of apical and basal neural progenitor cells. Sox2+ (apical neural progenitor cells: green) and Tbr2+ (basal progenitor cells: red) were detected in coronal sections of WT (A) and Heterozygous *Hif1α* mutant (Het, B) telencephalon at E14.5. Scale bars; 50 μm. Three independent experiments are performed and one representative image is shown, respectively. The number of Sox2+ (C) and Tbr2^+^ (D) cells was counted. Gray bar; WT, black bar; Het. Data are mean ± S.E.M of 6 sections. Statistical differences were assessed with Student’s *t*-test.

**Additional file 4: Figure S4.** Conditional ablation of *Hif1α* in neuroepithelial cells impairs vascular network formation in the telencephalon. IB4-FITC+ blood vessels (magenta) were detected in coronal sections of WT (A and C) and KO (B and D) telencephalon at the indicated embryonic stages. Higher magnification images of the area enclosed by rectangular dotted-line are shown as insets. Scale bars; 200 μm. Four independent experiments are performed and one representative image is shown, respectively. The area of blood vessels was measured by Image J, and the relative values are presented as bar graph (E). White bar; WT, gray bar; KO. Data are mean ± S.E.M of 5 sections. Statistical differences were assessed with Student’s *t*-test, * *p* < 0.05. IB4-FITC+ blood vessels (green) and cleaved caspase3+ apoptotic cells were detected in coronal sections of WT (E) and KO (F) telencephalon at E18.5. Regressing vessels are indicated by arrow heads. Scale bars; 100 μm.

**Additional file 5: Figure S5.** Overexpression of sFlt1 does not affect vascular network formation in the telencephalon. sFlt1-pCI (sFlt1) plasmid was electroporated into neural progenitor cells in one side of telencephalic semisphere at E13.5 (Ipsilateral: C and D). Another side of telencephalic semisphere is used as control (Contralateral: C and D). IB4-FITC+ blood vessels (green) and transfected cells (magenta) were detected in coronal sections of the cortex at E18.5. Scale bars; 100 μm. Three independent experiments are performed and one representative image is shown, respectively.

## Data Availability

The datasets used and /or analyzed during the current study are available from the corresponding author on reasonable request.
