# Supplementary figures and images for: Hif1α-dependent hypoxia signaling contributes to the survival of deep-layer neurons and cortex formation in a mouse model
Source: Mol Brain. 2022 Mar 31;15:28. doi: 10.1186/s13041-022-00911-0 (PMC8973788; doi:10.1186/s13041-022-00911-0)

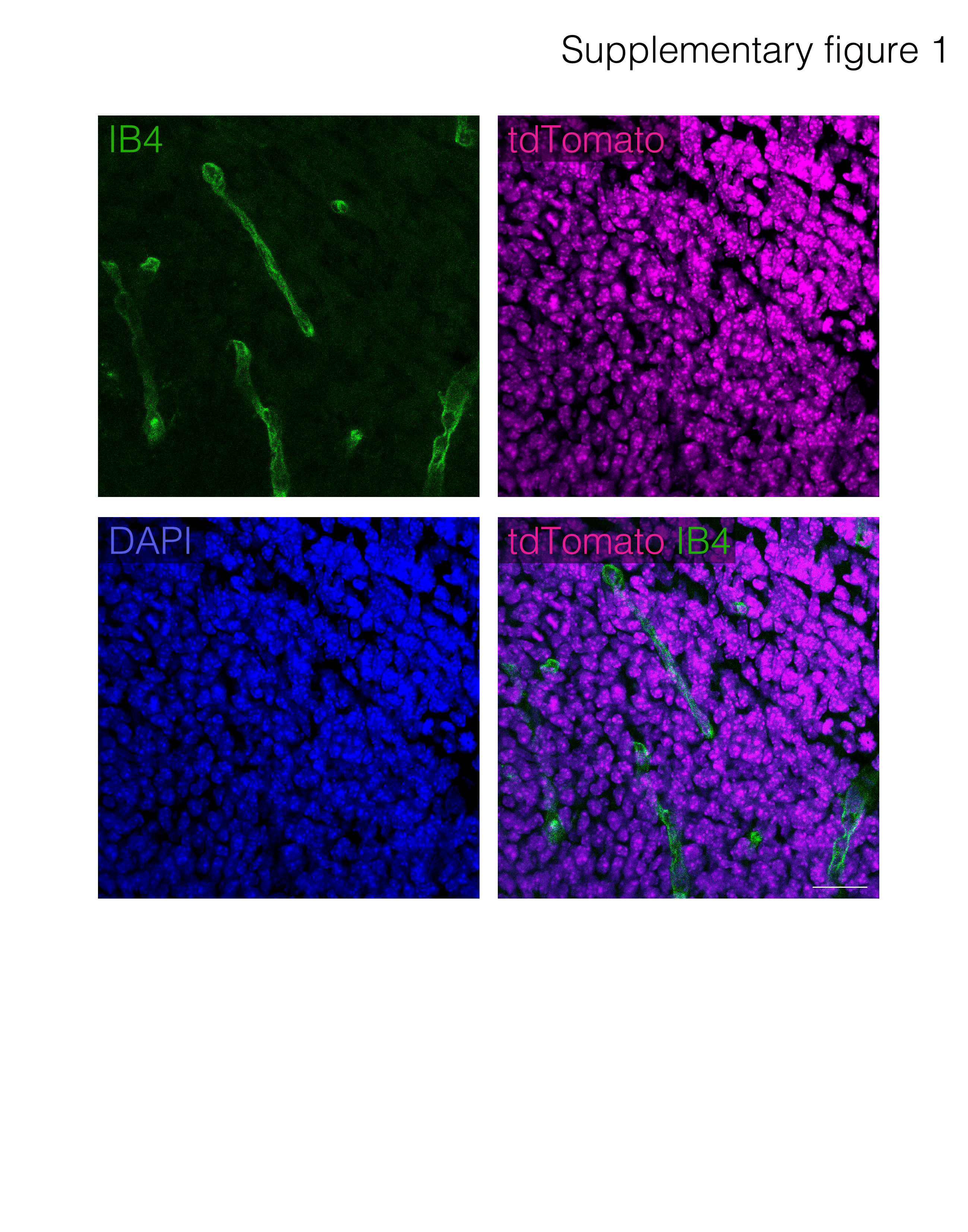

Supplement: Supplementary file 1 — Additional file 1: Figure S1. Cre is activated in neural progenitor cells and neural cells. IB4-FITC-positive blood vessels (green), tdTomato-positive cells (magenta), and nucleus (blue) were detected in coronal sections of Sox1-Cre+/−; ROSA26/CAG-floxed STOP-tdTomato+/− telencephalon at E16.5. Conforcal optical slices were collected and maximum-intensity projections of 15 μm stacks were made. Scale bars; 10 μm. Three independent experiments are performed and one representative image is shown, respectively. [file 13041_2022_911_MOESM1_ESM.tif]

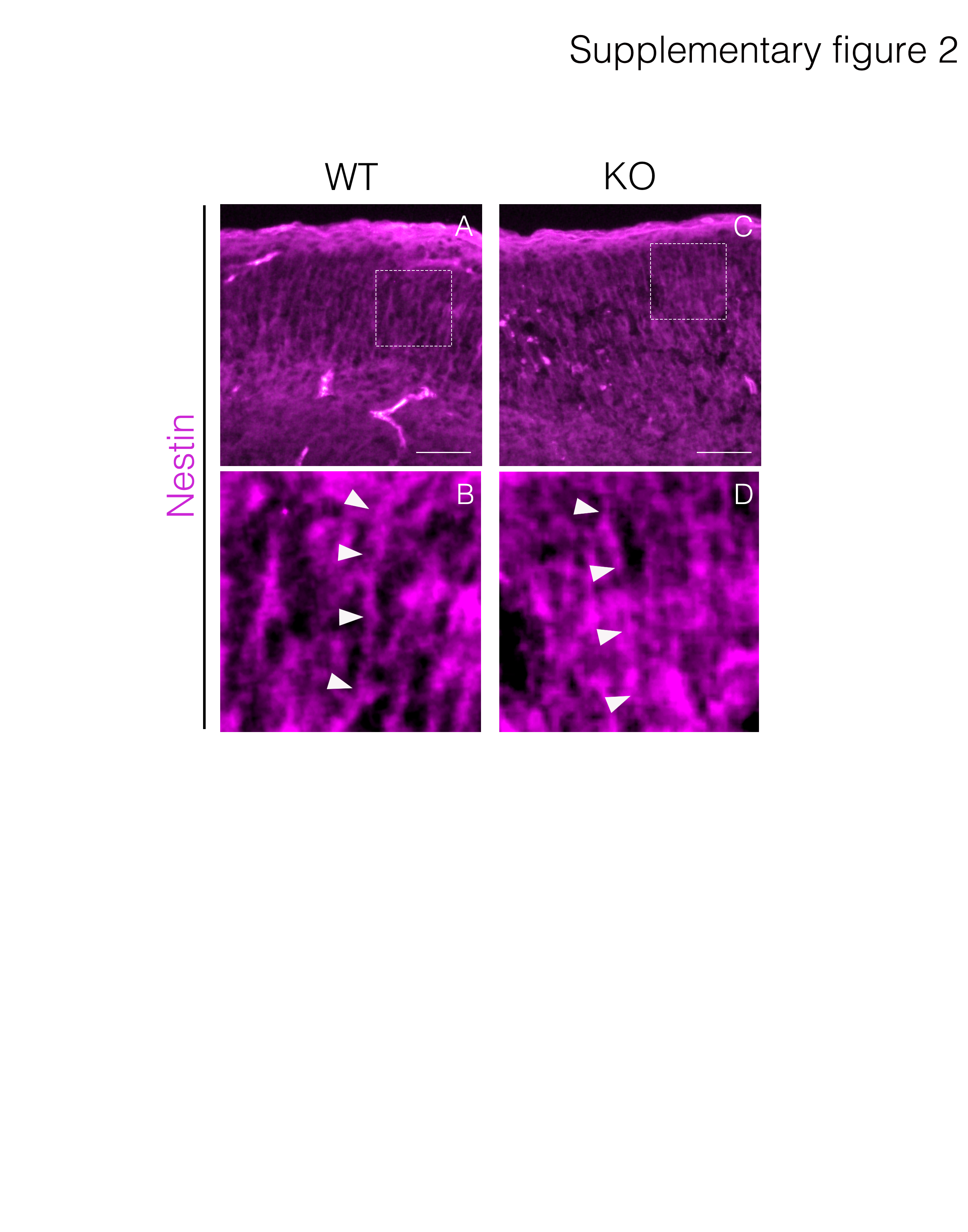

Supplement: Supplementary file 2 — Additional file 2: Figure S2. Radial glial fibers are normally formed in KO telencephaon. Morphology of radial glial cell fibers were analyzed by immunofluorescence using anti-Nestin antibody in coronal sections of WT (A and B) and KO (C and D) telencephalon at E13.0. B and D are magnifications of the region enclosed with dotted-line in A and C, respectively. White arrowheads indicate a radial glial fiber. Scale bars; 100 μm. Five independent experiments are performed and one representative image is shown, respectively. [file 13041_2022_911_MOESM2_ESM.tif]

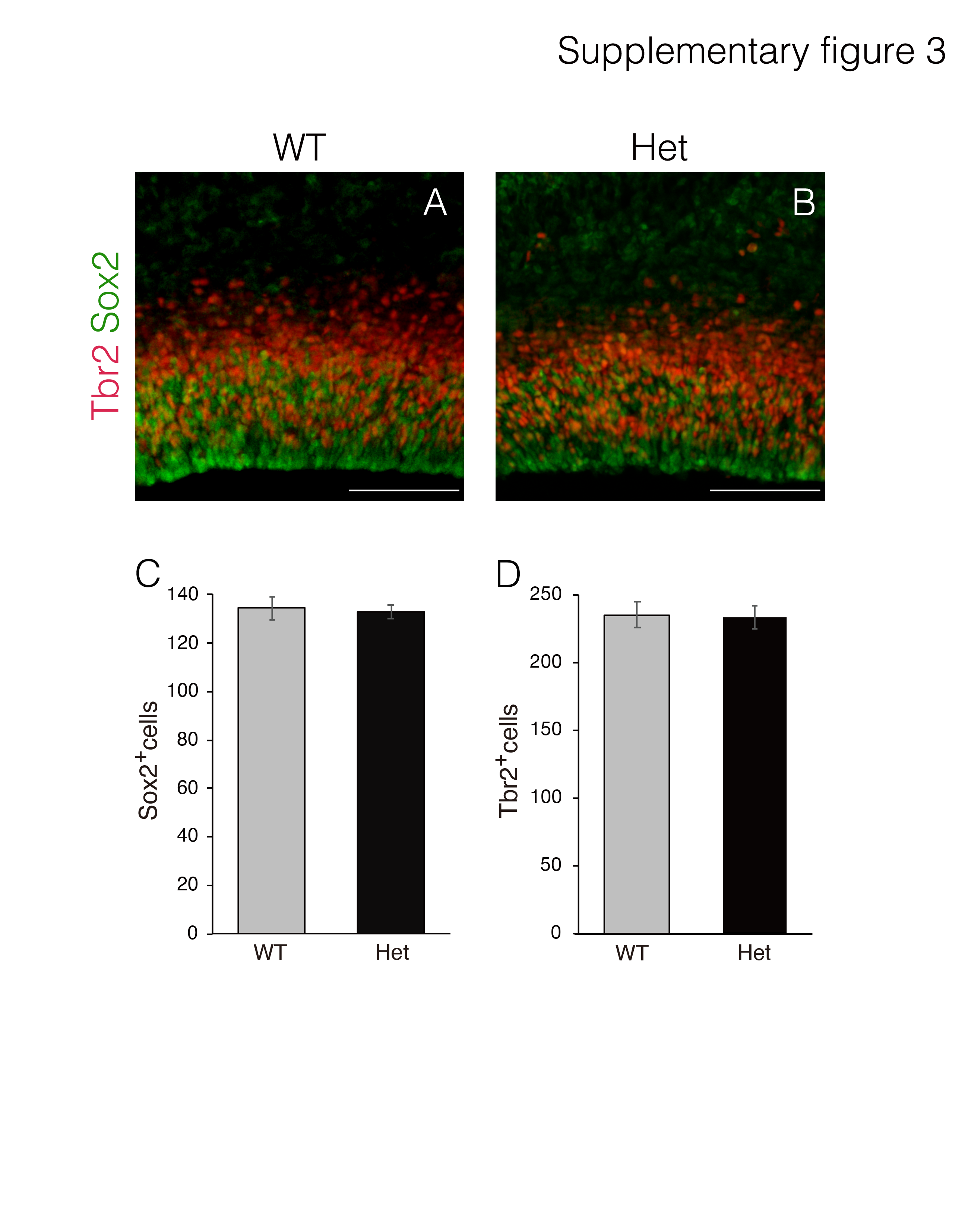

Supplement: Supplementary file 3 — Additional file 3: Figure S3. Heterozygous Hif1α ablation does not affect the development of apical and basal neural progenitor cells. Sox2+ (apical neural progenitor cells: green) and Tbr2+ (basal progenitor cells: red) were detected in coronal sections of WT (A) and Heterozygous Hif1α mutant (Het, B) telencephalon at E14.5. Scale bars; 50 μm. Three independent experiments are performed and one representative image is shown, respectively. The number of Sox2+ (C) and Tbr2+ (D) cells was counted. Gray bar; WT, black bar; Het. Data are mean ± S.E.M of 6 sections. Statistical differences were assessed with Student’s t-test. [file 13041_2022_911_MOESM3_ESM.tif]

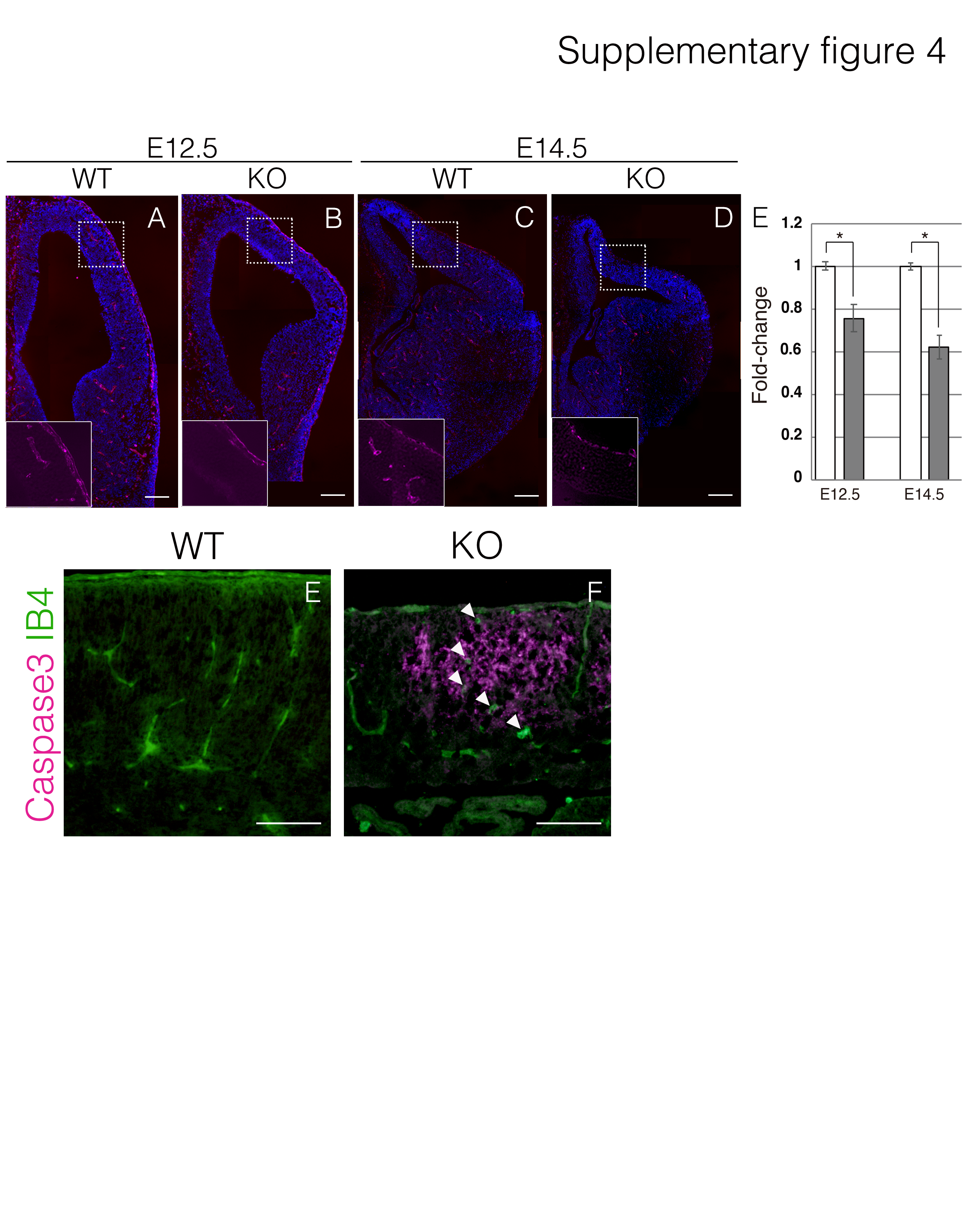

Supplement: Supplementary file 4 — Additional file 4: Figure S4. Conditional ablation of Hif1α in neuroepithelial cells impairs vascular network formation in the telencephalon. IB4-FITC+ blood vessels (magenta) were detected in coronal sections of WT (A and C) and KO (B and D) telencephalon at the indicated embryonic stages. Higher magnification images of the area enclosed by rectangular dotted-line are shown as insets. Scale bars; 200 μm. Four independent experiments are performed and one representative image is shown, respectively. The area of blood vessels was measured by Image J, and the relative values are presented as bar graph (E). White bar; WT, gray bar; KO. Data are mean ± S.E.M of 5 sections. Statistical differences were assessed with Student’s t-test, * p < 0.05. IB4-FITC+ blood vessels (green) and cleaved caspase3+ apoptotic cells were detected in coronal sections of WT (E) and KO (F) telencephalon at E18.5. Regressing vessels are indicated by arrow heads. Scale bars; 100 μm. [file 13041_2022_911_MOESM4_ESM.tif]

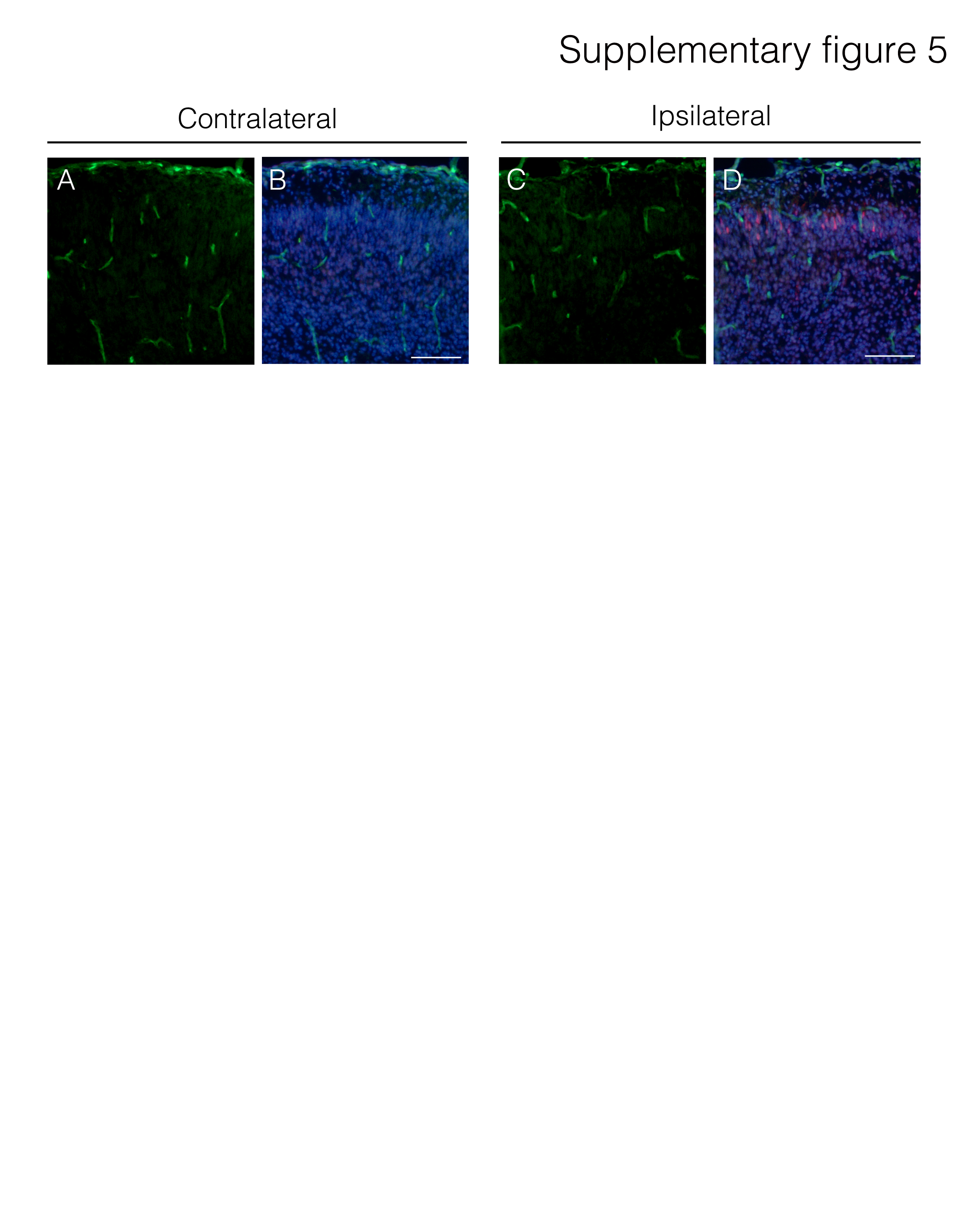

Supplement: Supplementary file 5 — Additional file 5: Figure S5. Overexpression of sFlt1 does not affect vascular network formation in the telencephalon. sFlt1-pCI (sFlt1) plasmid was electroporated into neural progenitor cells in one side of telencephalic semisphere at E13.5 (Ipsilateral: C and D). Another side of telencephalic semisphere is used as control (Contralateral: C and D). IB4-FITC+ blood vessels (green) and transfected cells (magenta) were detected in coronal sections of the cortex at E18.5. Scale bars; 100 μm. Three independent experiments are performed and one representative image is shown, respectively. [file 13041_2022_911_MOESM5_ESM.tif]
